# Clinical Cohort Study in Canine Patients, to Determine the Average Platelet and White Blood Cell Number and Its Correlation with Patient’s Age, Weight, Breed and Gender: 92 Cases (2019–2020)

**DOI:** 10.3390/vetsci8110262

**Published:** 2021-11-03

**Authors:** Isabel de Marcos Carpio, Anita Sanghani-Kerai, Miguel A. Solano, Gordon Blunn, Alexandra Jifcovici, Noel Fitzpatrick

**Affiliations:** 1 Fitzpatrick Referrals, Surrey GU7 2QQ, UK; miguels@fitzpatrickreferrals.co.uk (M.A.S.); ale.jifcovici@gmail.com (A.J.); noelf@fitzpatrickreferrals.co.uk (N.F.); 2 School of Pharmacy and Biomedical Sciences, University of Portsmouth, Portsmouth PO1 2DT, UK; gordon.blunn@port.ac.uk

**Keywords:** platelet-rich plasma, regenerative therapy, platelets, white blood cells, canine, degenerative, joint, age, sex, weight

## Abstract

Due to its easy preparation and that it is well tolerated, the use of autologous platelet-rich plasma (PRP) has become increasingly popular in regenerative medicine. However, there are still no clear guidelines on how it should be classified or whether the individual canine patient’s clinical status can influence its quality. Objective: This study aims to show if the weight, age, sex, neutered status or breed of canine patients have any correlation with the composition of PRP. Design: A blinded count of the platelets and white blood cells (WBC) was performed from 111 samples from 92 client owned dogs undergoing treatment for degenerative joint disease (DJD). The results were analysed using Pearson correlation test, ANOVA test or Student *T*-test. Results: There is a positive correlation between the number of platelets and WBC in canine patients of different breeds, but there was no significant difference on the platelet number and WBC number among the different breeds. The weight of the patient is also directly correlated to the platelet number (*p* = 0.003) but not WBC number. WBC number was negatively correlated to the weight of the patient. The sex and age of the patient did not affect platelets and WBC number, although WBC number is increased in non-neutered male population (*p* = 0.003). However, it would be interesting to investigate whether the growth factors released from the platelet granules are affected by patient variables in a canine population. Conclusions: Our results show that it is possible to obtain good quality autologous PRP, irrespective of age, sex, neutered status or weight of the patient, for PRP regenerative therapy.

## 1. Introduction

Platelet-rich plasma (PRP) is plasma enriched with a platelet concentration five times above the baseline concentration normally contained in whole blood [1,2,3]. PRP can be prepared easily, is well tolerated and very rarely leads to complications [4], making it attractive in human and veterinary medicine. In human medicine, PRP has been used in the fields of cardiology, dental and maxillofacial [1,5,6,7,8]. In musculoskeletal injuries, PRP has been used in human and veterinary medicine for different pathologies, such as osteoarthritis (OA), and injuries of tendon, muscle, cartilage and bone [4,9,10,11,12,13,14,15,16,17,18,19,20].

Platelets encourage tissue regeneration by inducing cell recruitment, proliferation and differentiation [4,21]. They contain granules that release growth factors, which act individually or synergistically to promote tissue healing. These growth factors include platelet-derived growth factor (PDGF), transforming growth factor-B1 (TGF-B1),transforming growth factor-B2 (TGF-B2), vascular endothelial growth factor (VEGF), basic fibroblastic growth factor (bFGF) and epidermal growth factor (EGF) [2,21,22,23,24,25]. They also stimulate the migration of other cells in the body to the areas of administration promoting healing of the damaged tissue.

Platelets are not the only important components of a PRP. The presence or absence of mononuclear cells, neutrophils and red blood cells have been reported to affect the clinical efficacy of the product and the inflammatory response after PRP injections [22]. To date, there is no consensus on the best process to prepare PRP or the optimal concentration of blood components to include in the product [1].

Standardising the PRP is important and platelets can be classified according to the absolute platelet concentration, the method of activation and the presence or absence of WBC and neutrophils relative to the base line [26]. More recently, the PRP has been classified by the dose of injected platelets, the efficiency of production, the purity and the activation method of the PRP which has been used [4,27].

However, none of these methods have been formally adopted in either human or veterinary medicine and the description of PRP is ambiguous and poorly defined [4]. Furthermore, depending on the system used to produce the PRP, the amount of blood that needs to be collected can vary from 6 mL to 120 mL, and whilst there is a positive correlation between the platelet concentration with volume of blood and the centrifugal force used for isolation, there is no correlation between the volume of PRP and platelet concentration [28]. The optimal WBC concentration in PRP is still debatable. This is because WBC have antimicrobial properties, but when used intraoperatively, WBC can increase acute pain and its pro-inflammatory effect can be detrimental for articular cartilage healing [22,29]. The optimal concentration and ratio of platelets and WBC in PRP is unknown [1]. Human clinical trials have shown that the use of PRP improves pain in the short and medium term [4] better than other intra-articular (IA) injections, such as Hyaluronic Acid (HA). The aim of this large clinical cohort study is to determine what the average platelet concentration in canine PRP is and what differences exist between weight, age, gender and breed. At the same time, we aim to determine what is the WBC concentration within the samples and if there are any differences between weight, age, gender and breeds. 

## 2. Materials and Methods

### 2.1. Patient Recruitment

The patients included in this study were recruited from 2019 and 2020, attending the hospital for palliative treatment of osteoarthritis in different joints. None of these dogs were on steroids. All dogs were on standard canine painkillers, such as Gabapentin, Paracetamol, amantadine and non-steroidal anti-inflammatory drugs (NSAIDS). 

A total of 111 dogs were included in the study. The median age was 88.1 months and median weight was 39.1 kg. During the period of the study, 111 samples were collected from 92 patients. There were 21 breeds represented and two groups of crossbreeds, one with an unknown origin and a second one with Labrador crossbreeds. The remaining dog breeds included 30 Labrador Retrievers, 11 Staffordshire Bull Terrier, 8 German Shepherd Dogs, 5 Golden Retrievers, 5 English Springer Spaniel, 3 Cocker Spaniels, 3 English Bulldogs, 2 Chow Chows, 1 Basset Hound, 1 Dachshund, 1 Rhodesian Ridgeback, 1 Miniature Schnauzer, 1 Hungarian Vizsla, 1 Bearder Collie, 1 Border Collie, 1 Shih Tzu, 1 Boxer, 1 Jack Russel Terrier, 1 Russian Terrier, 1 Flat Coat Retriever and 13 crossbreed dogs; among which there were 8 of unknown origin and 5 Labrador crossbreeds. Among our patients, there were 37 females, 33 neutered and 4 entire, and 72 males, 48 neutered and 26 entire.

With the informed consent of the owners, blood was obtained from dogs undergoing intra-articular injection as treatment for degenerative joint disease (DJD). This was part of a multimodal therapy where PRP or PRP and stem cells or PRP and Hyaluronic acid was used. A complete clinical evaluation was performed, including haematology and serum biochemistry. 

For all these patients, PRP and/or stem cells injection had been prescribed as part of their treatment protocol. We assessed a small aliquot of this PRP without compromising the sample for treatment. This study was unable to collect PRP in patients where there was a limited amount for injection. The following information was obtained from the patient file: age, breed, body weight at the time of injection and gender.

### 2.2. Blood Extraction and PRP Collection

The volume of blood was drawn depending on the patient’s body weight; patients over 10 kg of body weight had 18 mL of blood extracted, whereas patients less than 10 kg of body weight had 9 mL of blood extracted. This decision was made on the principle that we can only extract 10% of blood body volume. All dogs were sedated with a standard sedation protocol consisting of Medetomidine (Sedator 1.0 mg/mL solution for injection, Dechra Pharmaceuticals, Northwich, UK) 5 mcg/kg to 10 mcg/kg IV and Methadone (Comfortam 10 mg/mL solution for injection, Dechra Pharmaceuticals, Northwich, UK) 0.2 mg/kg IV. A large fur clip was made on the anatomic location of one of the jugular veins and it was prepared aseptically. Blood samples were collected into a syringe containing 10% of citrate anticoagulant (ACD-A, Anticoagulant Citrate Dextrose Solution, Biomet Biologics, Warsaw, IN, USA).

The blood was collected in aseptic conditions by licensed veterinary nurses (RVNs) using a 21 G butterfly needle and a 10 or 20 mL syringe, depending on the blood volume to be extracted, that contained 10% ACD-A. The blood was centrifuged in sPRP tubes (NTL Biologica, Oxfordshire, UK) at 3300 rpm for 3 min to separate the PRP (buffy layer) from the RBC and plasma. A small aliquot (100 uL) of the PRP was then assessed for the number of WBC and platelets using a light microscope (Olympus, Tokyo, Japan). The rest of the PRP was then injected into the joints of canine patients. 

All the platelet counts were performed by the same person. No information of age, weight, gender or breed about the patients was given to this person, apart from the amount of PRP obtained from each patient, prior to performing the count. A quantity of 0.01 mL of the platelet sample was diluted and mixed thoroughly with 4 mL of saline. Of this volume, 0.1 mL was mixed with an equal volume of trypan blue and the number of platelets were counted using a haemocytometer under a light microscope (Olympus) at 40× magnification.

To obtain the number of WBC in each patient, 20 μL of PRP sample was mixed with 20 μL of trypan blue and the WBC were counted using a haemocytometer under a light microscope (Olympus) at 20× magnification. The final number was obtained using the following formula, *N* = *m* × *d* × *v* × 10^4^, in which, “*N*” is the total number of WBC, “*m*” is the number of WBC counted in the hematocytometer, “*d*” is the dilution of the sample and “*v*” is the total volume of the PRP sample in millilitres (mL). 

### 2.3. Statistical Analysis

The data in this study was assessed for normal distribution using a Kolmogorov–Smirnov test. A Pearson’s correlation test was used to determine the correlation between the data. All data were statistically evaluated using SPSS statistical software 25 (IBM Corp., Armonk, NY, USA). If the data were parametric (normally distributed), the results were presented as mean ± Standard deviation (SD) and statistical comparison was carried out using an independent sample *t* test. If the data were non-parametric, the results were presented as median (Range) and analysed using a Mann–Whitney U test. *p* values of <0.05 were considered to be statistically significant.

## 3. Results

### 3.1. Platelet Number vs. WBC Number

**Figure 1:**
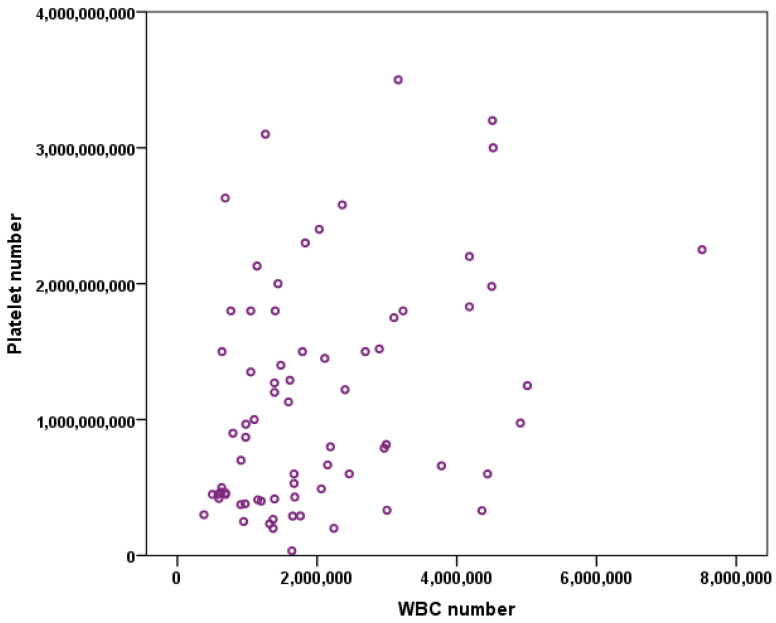
Pearson’s test show a positive correlation (*p* = 0.02, r = 0.357) between platelet count and white blood cells (WBC) count across all the dog breed included in our study.

Pearson’s test showed that there is a strong positive correlation between the platelet number and the WBC number (*p* = 0.02, r = 0.357) (Figure 1).

### 3.2. Platelets and WBC vs. Age

**Figure 2:**
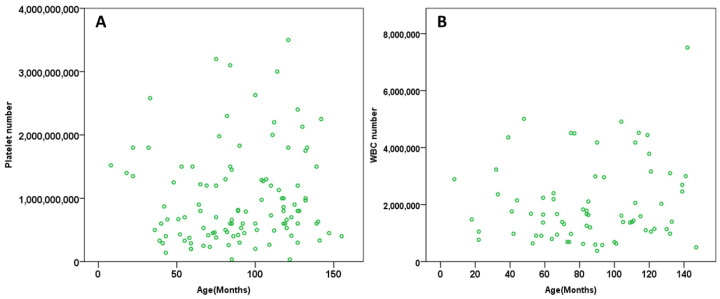
The scatter dot plot demonstrating the relationship between platelet number and age of the patient (months). In relation to the age of the patient, Pearson´s test did not show any correlation on platelet count (*p* = 0.465, r = 0.07) (**A**) and WBC count (*p* = 0.283, r = 0.141) (**B**) across all the patients included in this study.

Pearson’s correlation demonstrated that there was no significant correlation between the age of the patient and platelet number (*p* = 0.465, r = 0.07) or WBC number (*p* = 0.283, r = 0.141). The number of platelets or WBC obtained was not dependent on patient age (Figure 2).

### 3.3. Platelets and WBC Number vs. Weight

**Figure 3:**
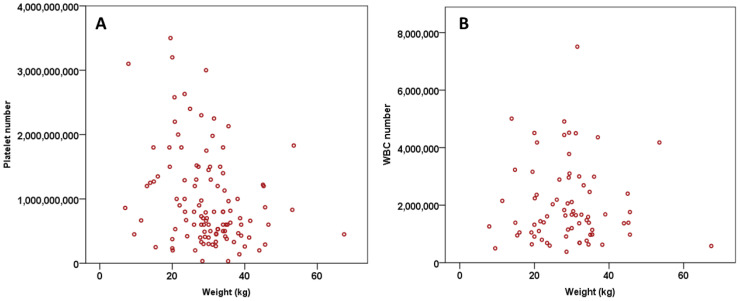
The scatter dot plot demonstrating the relationship of platelet number with weight of the patient (kg). Pearson´s test showed a negative correlation between weight of the patient and platelet count (*p* = 0.003, r = −0.283) (**A**) and with WBC count (*p* = 0.873, r = −0.019) (**B**).

Pearson’s correlation showed that the platelet number was negatively correlated to the weight of the patient (*p* = 0.003, r = −0.283) and this was significant. There was also a negative correlation between the WBC number and the weight of the patient (*p* = 0.873, r = −0.019). Even though these results imply that smaller breeds have lower platelet numbers, due to the wide range of weight within a breed, it is difficult to predict a correlation between size of the patient within a specific breed and platelet count (Figure 3).

### 3.4. Platelets and WBC vs. Breed

To determine if there is a difference between platelet or WBC number with the breed of patients in this study, an ANOVA test was used. There was no significance difference between them. Nevertheless, platelet count was very close to 0.05.

Platelet number *p* = 0.049.

White blood cell number: *p* = 0.219.

### 3.5. Platelets and WBC vs. Sex

**Figure 4:**
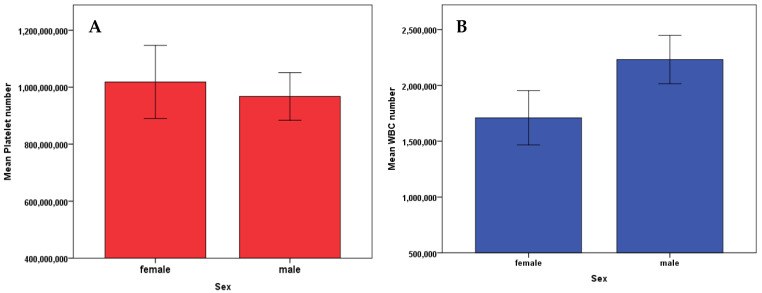
**A** two-tailed *t*-test showed no difference between sex of the patient and platelet count (*p* = 0.736) (**A**), or WBC number (*p* = 0.126) (**B**). Error bars represent ± 1 Standard error.

To check for statistical difference between platelet or WBC number with the sex of the animal across all breeds, we performed an independent sample *t*-test (Two tailed). We found that there was no significant difference for platelet number (*p* = 0.736) and WBC (*p* = 0.126) between male and female dogs (Figure 4). 

**Figure 5:**
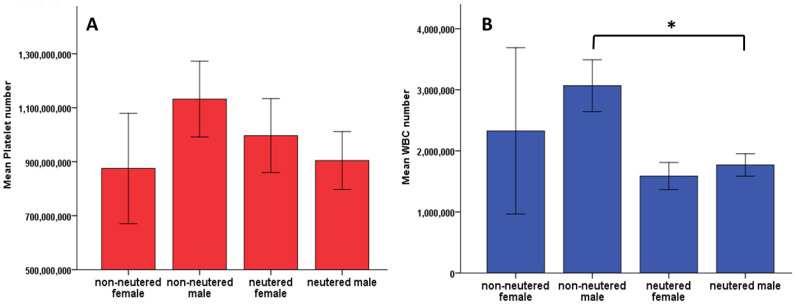
Student’s *t*-test showed no statistical difference between patient’s neutered status and platelet number (*p* = 0.765) (**A**). However, there was a significant difference in WBC number between neutered and non-neutered patients (*p* = 0.003), showing increased number in the non-neutered male group (**B**). * represents significance *p* < 0.05. Error bars represent ± 1 Standard error.

Nevertheless, when comparing the neutered status of our patients with platelet and WBC number, there was a significant difference between WBC concentration and the patient’s neutered status in the male group (*p* = 0.003) (Figure 5).

## 4. Discussion

The results of this clinical study show that there is a positive correlation between the number of platelets and WBC in canine patients of different breeds (Figure 1), but there was no significant difference between the platelet number (*p* = 0.049) and breed or WBC number (*p* = 0.219) and breed of the patient. The weight of the patient is also directly correlated to the platelet number but not WBC number (Figure 3). WBC number was negatively correlated to the weight of the patient (Figure 3B). Additionally, there was no correlation of the patient’s sex to the platelet and WBC number (Figure 4). The platelet number or WBC number did not differ with age of the patient (Figure 2). Furthermore, the number of platelets did not vary at different time points for the same patient.

PRP has the potential to repair tissues with poor healing capability; therefore, it is increasingly being used clinically for the treatment of various soft and hard tissues [1,2,18,19,20,21,23,24,25]. The process of tissue repair is mediated by cytokines and growth factors, and in platelets this is present in the alpha granules [1,21,30,31]. Leukocytes, on the other hand, have a role in tissue repair, but their high concentrations can be counterproductive for tissue healing [1,21,32,33,34].

Our results demonstrate that the age of the canine patients had no effect on the number of platelets or WBC (Figure 2). This is similar to a study by Fujiwara et al. whereby 44 healthy Beagles were separated in different age groups to compare the effects of age on their blood cells. It was found that younger and middle-aged dogs had higher concentrations of Interleukin-2, Interleukin-2Ralfa and interferon-gamma than older dogs, but their red blood cell (RBC) and WBC count did not differ [35]. However, Bourgès-Abella et al., 2015, showed that in Beagles, platelet count increases with age and WBC count decreases [36]. A study by Harper et al. 2003, showed that WBC decreased with age in a study comparing the different age groups of two breeds, Beagle and Labrador. There was a strong difference detected within these two breeds in dogs among the age of 1 and 8 years old, with Beagles having significantly higher WBC counts than Labrador Retrievers [37].

A human study by Xiong and co-workers, 2018, showed that the platelet count in whole blood did not differ by age and sex in healthy patients. However, interestingly, even though the platelet count did not vary with age and sex, the growth factors released from the platelets were significantly different between male and female patients [38]. Similarly, Evanson et al. found no differences on platelet count between sex and age, but they found higher concentrations of growth factors in female patients compared to male patients and in patients older and younger than 25 years [39]. Mean platelet volume (MPV) has been recognised as a new predictor for hip OA in human medicine together with blood platelet–lymphocyte ratio (PLR) [40]. This means that the higher the concentration of these biomarkers in the serum of the patients, the higher the grade of OA. There is a strong correlation between age and severity of OA (*p* < 0.001), assuming that older patients would have a higher OA grade and thus a higher concentration of MPV and PLR in serum [40]. In our study, age does not have a correlation with platelet count or WBC. This could be because no data on the severity of OA in the patients recruited were collected. In veterinary medicine, severe OA is often present in relatively young patients due to orthopaedic diseases that were either diagnosed too late to have a surgical option of treatment, or the owners decided to have it treated medically. Therefore, correlation of age with severe OA is dependent on the joints affected and the pathology underneath.

Although there are no differences on PLT number or WBC depending on the sex of our patients (Figure 4), Bourgès-Abella et al. 2015, showed that platelet count was higher in females than males [36]. Nemeth et al. 2010, also showed a higher PLT count in females compared to males, and no statistical differences in WBC among sex [41]. However, we showed a significant difference in WBC number in non-neutered males compared with neutered males (Figure 5B).

Bourgès-Abella et al. 2015, also indicated that the weight of the patients neither correlated with platelet count nor WBC count. However, a study in human patients investigating the blood count of a group of people with different body mass index (BMI), showed a strong positive correlation between the BMI and WBC count. Similarly, this study shows a strong correlation between BMI and platelet count [42]. A high BMI could give an indication of the body fatness of a person. In veterinary medicine, the index used is the body condition score (BCS), which is a subjective number assigned to a dog based on a few key locations of its body. Unfortunately, despite our study results being similar to Furuncuoglu et al. by demonstrating a positive correlation of patient weight and platelets (Figure 3A), we did not collect the BCS of our patients [42].

However, it is difficult to compare results between different studies because PRP preparations are not consistently defined and PRP is prepared using different methods [28,43,44]. The results could also be different due to the different composition of leukocytes and platelets in the final PRP. Furthermore, PRP composition depends on the patient’s clinical status and blood composition. Importantly, a platelet from one individual will not contain the same amount of cytokines as a platelet from another individual; therefore, this presents differences between the platelets from every patient.

This study has several limitations. Firstly, the patient number in each breed is unequal. Even though we found that there is a statistically insignificant difference in platelet number and WBC number across the breed, the different types of breed included in this study open the possibility of having differences among them. We believe that this should not have a big impact on our results because the breeds included do not have any known haematological differences from the rest, such as Greyhounds, for example, do [45,46,47]. Another limitation was the lack of a body condition score for each of our patients. Among patients with the same weight, they could easily have a different body condition score depending on their size. Lastly, we did not grade the OA severity within a certain breed in an age group. Within a certain age group in a specific breed, dogs may have different degrees of OA. This could be due to diseases of genetic origin, such as elbow or hip dysplasia, or to genetic abnormalities in the blood, as is the case of rheumatoid arthritis [20,48,49,50].

Additionally, in our study, the platelet and WBC counts from whole blood was not compared to the PRP we isolated using the sPRP tubes. This could be interpreted as a limitation, although we do not believe this to be relevant. In a study by Evanson et al. [39] red blood cells and WBC were greatly reduced in PRP preparations, and platelet number was higher than in whole blood, as per its definition [1,2,51]. The platelet number in whole blood could be altered due to the formation of clumps during its analysis in the haematology machine. To obtain PRP, our blood samples were processed in an identical manner in order to eliminate potential variability in the results.

In conclusion, the results in our study show that there is a strong correlation between the weight of our patients and the platelet count in their PRP samples (Figure 3A). Even though there is no correlation with the other variables that we have studied with the platelet count or the white blood cells, the correlation of these variables with the growth factors released from the platelet granules is still unknown; as it has been shown in previous human studies [37,38], there could be differences between gender and age regarding the production of growth factors in the site injected with PRP. This study is clinically important as it is a cohort study including several common canine breeds and these results show that it is possible to obtain good quality autologous PRP, irrespective of age, sex, neutered status or weight of the patient, for PRP regenerative therapy.

## Data Availability

The data presented in this study are available on request from the corresponding author. The authors hold an electronic file of the patient data included in this study. Personal data about the patients are anonymised when used in any form of publications and presentations. All personal data about every dog are kept strictly confidential and does not leave the hospital.
